# *TMEM106B* Acts as a Modifier of Cognitive and Motor Functions in Amyotrophic Lateral Sclerosis

**DOI:** 10.3390/ijms23169276

**Published:** 2022-08-17

**Authors:** Arianna Manini, Antonia Ratti, Alberto Brusati, Alessio Maranzano, Isabella Fogh, Silvia Peverelli, Stefano Messina, Davide Gentilini, Federico Verde, Barbara Poletti, Claudia Morelli, Vincenzo Silani, Nicola Ticozzi

**Affiliations:** 1 Neurology Residency Program, Università degli Studi di Milano, 20122 Milan, Italy; 2 Department of Neurology and Laboratory of Neuroscience, IRCCS Istituto Auxologico Italiano, 20149 Milan, Italy; 3 Department of Medical Biotechnology and Molecular Medicine, Università degli Studi di Milano, 20122 Milan, Italy; 4 Dipartimento di Scienze del Sistema Nervoso e del Comportamento, Università degli Studi di Pavia, 27100 Pavia, Italy; 5 Department of Basic and Clinical Neuroscience, Maurice Wohl Clinical Neuroscience Institute, King’s College London, London SE1 8WA, UK; 6 Bioinformatics and Statistical Genomics Unit, IRCCS Istituto Auxologico Italiano, 20090 Milan, Italy; 7 Department of Pathophysiology and Transplantation, ‘Dino Ferrari’ Center, Università degli Studi di Milano, 20122 Milan, Italy

**Keywords:** amyotrophic lateral sclerosis, frontotemporal lobar degeneration, TMEM106B, alleles, cognition, motor neurons

## Abstract

The transmembrane protein 106B (*TMEM106B*) gene is a susceptibility factor and disease modifier of frontotemporal dementia, but few studies have investigated its role in amyotrophic lateral sclerosis. The aim of this work was to assess the impact of the *TMEM106B* rs1990622 (A–major risk allele; G–minor allele) on phenotypic variability of 865 patients with amyotrophic lateral sclerosis. Demographic and clinical features were compared according to genotypes by additive, dominant, and recessive genetic models. Bulbar onset was overrepresented among carriers of the AA risk genotype, together with enhanced upper motor neuron involvement and poorer functional status in patients harboring at least one major risk allele (A). In a subset of 195 patients, we found that the homozygotes for the minor allele (GG) showed lower scores at the Edinburgh Cognitive and Behavioral Amyotrophic Lateral Sclerosis Screen, indicating a more severe cognitive impairment, mainly involving the amyotrophic lateral sclerosis-specific cognitive functions and memory. Moreover, lower motor neuron burden predominated among patients with at least one minor allele (G). Overall, we found that *TMEM106B* is a disease modifier of amyotrophic lateral sclerosis, whose phenotypic effects encompass both sites of onset and functional status (major risk allele), motor functions (both major risk and minor alleles), and cognition (minor allele).

## 1. Introduction

Frontotemporal dementia (FTD) is one of the most common causes of early onset dementia, following Alzheimer’s disease (AD) and vascular dementia [1]. The spectrum of clinical phenotypes encompasses three subtypes, namely behavioral variant (bvFTD), semantic-variant primary progressive aphasia (svPPA), and nonfluent-variant PPA (nfvPPA) [2]. Neuropathological changes are represented by intranuclear and/or cytoplasmic accumulation of ubiquitinated proteins [3,4], mainly TAR DNA-binding protein 43 (TDP-43) [5,6] and, less frequently, hyperphosphorylated tau [7].

After the discovery that a non-coding hexanucleotide repeat expansion in the chromosome 9 open reading frame 72 (*C9orf72*) gene could result in either FTD, amyotrophic lateral sclerosis (ALS), and FTD/ALS phenotypes, with common TDP-43 neuropathology, it became clear that these disorders are part of the same disease continuum [8]. Beyond some established and distinct disease-causing genes, which account for a limited proportion of familial and, to a lesser extent, sporadic cases [i.e., progranulin (*GRN*), microtubule-associated protein tau (*MAPT*), tank-binding kinase 1 (*TBK1*), valosin containing protein (*VCP*), and charged multivesicular body protein 2B (*CHMP2B*) for FTD; TAR DNA binding protein (*TARDBP*), fused in sarcoma (*FUS*) and superoxide dismutase 1 (*SOD1*) for ALS] [7,9,10,11,12,13,14,15], most FTD and ALS forms show a still unclear multifactorial etiology. Therefore, over the last decades, genetic studies have focused on the identification of common variants which could explain at least part of the missing heritability of the FTD/ALS spectrum. In this scenario, a genome-wide association study (GWAS) performed in 2010 identified a novel susceptibility locus for FTD with cytoplasmic inclusions of TDP-43 on chromosome 7p21, which contains the transmembrane protein 106B (*TMEM106B*) gene [16]. *TMEM106B* encodes a transmembrane endosomal and lysosomal glycoprotein [17] expressed in neuronal, glial, and endothelial cells, whose impairment results in altered lysosomal structure and function [18].

The common allele (T or A, according to the considered strand) of the top single nucleotide polymorphism (SNP) rs1990622, together with those of other SNPs mapping at the same linkage disequilibrium block, have been associated with an increased risk of FTD, especially, but not exclusively, in the presence of TDP-43 pathology (risk allele) [16,19,20,21,22,23]. Although *TMEM106B*-related susceptibility to FTD was independent of the *GRN* mutation status of patients [16], the burden of FTD risk in *GRN*-mutated patients was higher compared to non-*GRN* carriers [16] and controls [20]. Further, the risk allele of rs1990622 was over-represented in FTD patients harboring *GRN* pathogenic mutations [23,24] and both in FTD and FTD/ALS patients with *C9orf72* expansions [22]. 

The major rs1990622 risk allele has been associated with both higher expression levels of *TMEM106B* in the brain and reduced plasma levels of Progranulin, encoded by the *GRN* gene [20]. Although the underlying mechanism is still debated [17,20,25,26,27], these observations support the presence of a common pathogenic cascade linking these two actors in FTD. Similarly, a direct link between TMEM106B and *C9orf72* has been recently proposed because siRNA-mediated gene silencing of *C9orf72* was able to rescue lysosomal size and acidification in vitro, which had been altered by TMEM106B overexpression [28].

In the last decade, the role of *TMEM106B* as a disease modifier in FTD and other neurodegenerative disorders, especially those characterized by TDP-43 pathology, has been widely investigated, with variable and sometimes contradictory results (i.e., enhanced cognitive deterioration associated with the major rs1990622 risk allele in FTD patients). To date, few studies have focused on the impact of *TMEM106B* on disease susceptibility and genotype-phenotype correlation in ALS cohorts, revealing no association of the rs1990622 SNP with age at onset or death but a doubtful protective role of the minor allele (C or G, according to the considered strand) on cognitive functions [29,30,31,32]. Therefore, the aim of this work was to further explore the influence of rs1990622 on several phenotypic traits of a large cohort of ALS patients, including age and site of onset, survival, upper and lower motor neuron signs, functional status, disease progression, cognitive and behavioral impairment.

## 2. Results

Our study cohort was composed of 865 Italian ALS patients, 554 (64.0%) of whom were males and 311 (36.0%) females (Table 1). A total of 20 patients (2.3%) had a positive family history of ALS, while the remaining were sporadic. The hexanucleotide repeat expansion in the *C9orf72* gene was present in 20 patients, while four had a mutation in *TARDBP*, two in *FUS,* and none in *SOD1*. The median age at onset was 61.6 (13.6–90.6) years, and the median survival was 27.5 (2.9–450.6) months. The site of onset, available for 815 patients, was bulbar in 206 cases (25.3%) and spinal in the remaining 609 (74.7%). The clinical scale for ALS severity, namely ALS Functional Rating Scale Revised (ALSFRS-R), was available for 449 patients, revealing a median value of 40 (9–48). Median Penn Upper Motor Neuron Score (PUMNS), employed to measure upper motor neuron signs, which had been reported for 690 patients, was nine (0–29). Amongst the 531 patients for whom the Medical Research Council (MRC) scale was available to assess the burden of lower motor neuron involvement, the median score was 54 (6–60). In order to investigate cognitive functions, the Edinburgh Cognitive and Behavioral ALS Screen (ECAS)—Italian version was performed on 195 patients, with a median score of 105 (31–129) for the total score, 78 (21–97) for the ALS-specific score, and 27 (10–34) for the ALS-non-specific score. According to the Strong revised criteria, 87 out of 195 patients (44.6%) could be classified as cognitively normal ALS (ALScn), 35 (17.9%) as behaviorally impaired ALS (ALSbi), 49 (25.1%) as cognitively impaired ALS (ALSci), and 24 (12.3%) as cognitively and behaviorally impaired ALS (ALScbi). According to the Rascovsky and Gorno-Tempini criteria for behavioral variant FTD (bvFTD) and PPA, respectively, 25 (2.9%) of our patients were affected by ALS/FTD. In order to investigate the behavioral profile of 155 patients, the Frontal Behavioral Inventory (FBI) was employed, showing a median total score of two (0–5), median A score of one (0–4), and median B score of zero (0–2). The demographic and clinical features of the cohort are reported in Table 1.

Genotype data of the rs1990622 SNP in the *TMEM106B* gene revealed that the minor allele frequency (G allele) was 0.40 within our cohort. Out of 865 patients, 305 (35.3%) were homozygous for the major allele (AA genotype), 421 (48.7%) were heterozygous (AG genotype), and 139 (16.1%) were homozygous for the minor allele (GG genotype) (Table 1). We also confirmed that rs1990622 is in linkage disequilibrium with the rs3173615 SNP in the coding region (p.T185S) of the *TMEM106B* gene (r_2_ = 0.976).

We found a significant association between the rs1990622 SNP and the site of disease onset under the dominant model (*p* = 0.023) (Table 2). Specifically, the proportion of patients with bulbar onset was significantly higher among carriers of the AA genotype compared to the AG and GG ones (30.0% in AA vs. 22.7% in (AG + GG), *p* = 0.023), whereas the rate of spinal onset was overrepresented among patients with the AG and GG genotypes compared to those homozygous for the major allele (77.3% in (AG + GG) vs. 70.0% in AA, *p* = 0.023) (Table S1).

**Figure 1:**
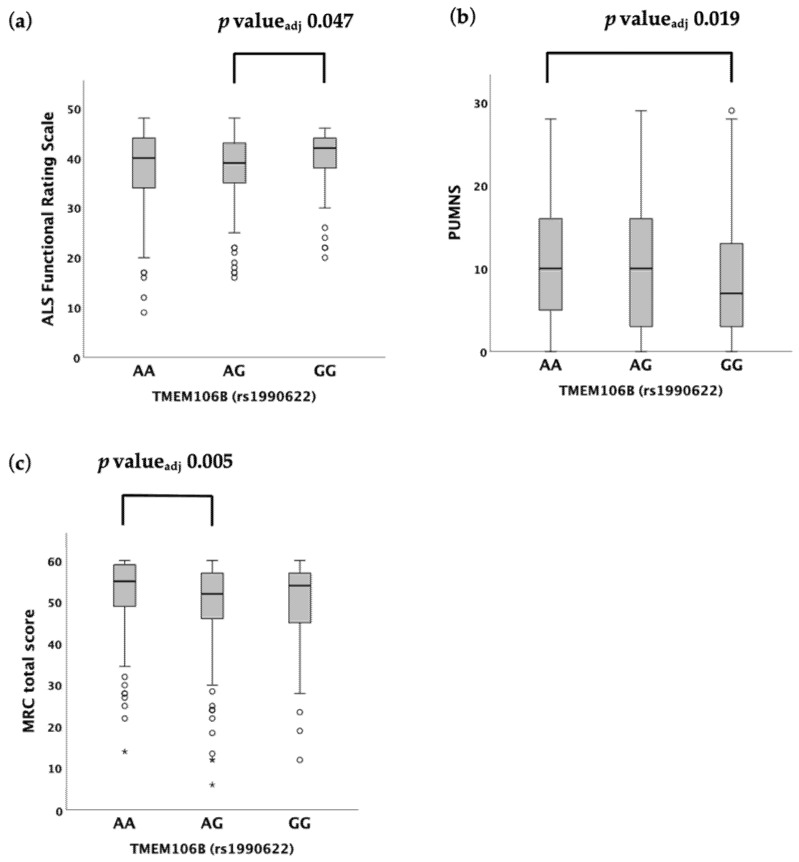
Distribution of variables amongst *TMEM106B* rs1990622 genotypes according to the Kruskal–Wallis one-way analysis of variance for independent samples under the additive model. (**a**) ALSFRS-R. (**b**) PUMNS. (**c**) MRC total score. For each group, the bold line shows the median, the gray box represents the interquartile range (IQR) and whiskers show the 5° and 95° percentiles. Empty circles and asterisks represent outliers.

When we assessed the functional status of patients, we found that rs1990622 was associated with the ALSFRS-R under both the additive (median value 40 (9–48) in AA vs. 39 (16–48) in AG vs. 42 (20–46) in GG, *p* = 0.042) (Table 2; Figure 1A) and recessive (median value 39 (9–48) in (AA + AG) vs. 42 (20–46) in GG, *p* = 0.041) (Table 2; Figure S1A) models. A post hoc analysis revealed statistically significant differences in ALSFRS-R scores between the AG and GG carriers (*p* = 0.016, *p*_adjusted_ = 0.047) (Figure 1A), but not between the AG and AA carriers (*p* = 0.139, *p*_adjusted_ = 0.418), nor between the AA and GG carriers (*p* = 0.237, *p*_adjusted_ = 0.712) (Table S2).

Similarly, the PUMNS was associated with rs1990622 under the additive (median value 10 (0–28) in AA vs. 10 (0–29) in AG vs. 7 (0–29) in GG, *p* = 0.025) and recessive (median value 10 (0–29) in (AA + AG) vs. 7 (0–29) in GG, *p* = 0.015) (Table 2; Figure S1B) models. Pairwise comparisons showed that the PUMNS was significantly higher in AA vs. GG carriers (i.e., more upper motor neuron signs) (*p* = 0.006, *p*_adjusted_ = 0.019) (Figure 1B), whereas no significant difference was found between the GG and AG carriers (*p* = 0.055, *p*_adjusted_ = 0.164), as well as between the AG and AA carriers (*p* = 0.230, *p*_adjusted_ = 0.690) (Table S2).

While no association was detected between the rs1990622 genotype and the MRC total score under the recessive model (*p* = 0.424), a significant difference was demonstrated by employing the additive (median value 55 (14–60) in AA vs. 52 (6–60) in AG vs. 54 (12–60) in GG, *p* = 0.005) and dominant (median value 55 (14–60) in AA vs. 53 (6–60) in (AG + GG), *p* = 0.001) models (Table 2; Figure S1C). According to the post hoc analysis, MRC total score was significantly reduced in AG carriers compared to AA carriers (*p* = 0.002, *p*_adjusted_ = 0.005) (Figure 1C), while the difference between the AG and GG genotypes (*p* = 0.788, *p*_adjusted_ = 1.000) was not significant, as well as between the GG and AA genotypes after correction for multiple comparisons (*p* = 0.035, *p*_adjusted_ = 0.106) (Table S2).

**Figure 2:**
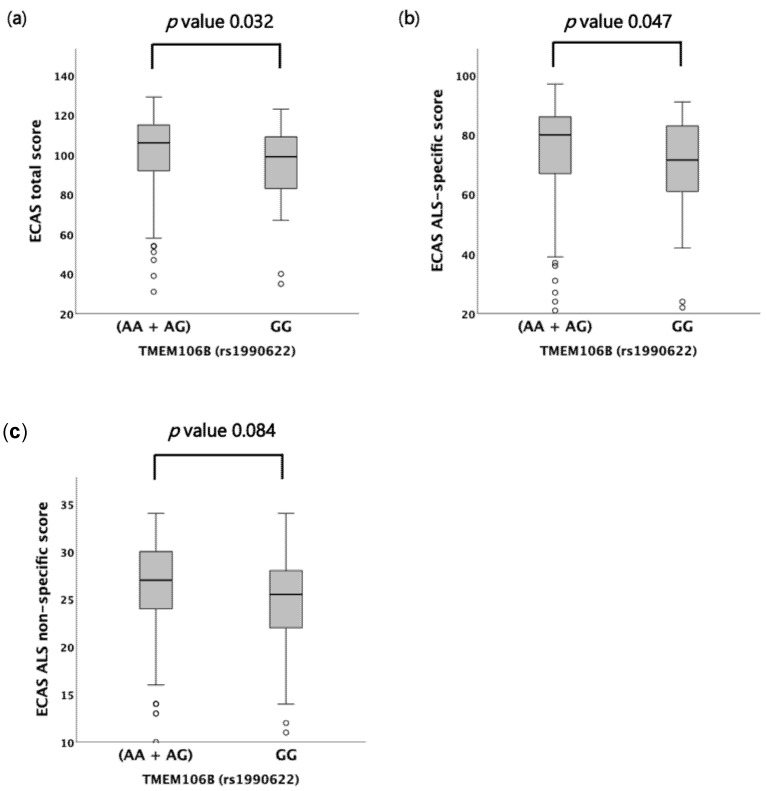
Distribution of ECAS total, ALS-specific and ALS-non-specific scores amongst *TMEM106B* rs1990622 genotypes according to the Kruskal–Wallis one-way analysis of variance for independent samples under the recessive model. (**a**) ECAS total score. (**b**) ECAS ALS-specific score. (**c**) ECAS ALS-non-specific score. For each group, the bold line shows the median, the gray box represents the interquartile range (IQR) and whiskers show the 5° and 95° percentiles. Empty circles represent outliers.

When we investigated the cognitive profile of a subset of patients, we found that the *TMEM106B* rs1990622 genotype influenced the ECAS total and ECAS ALS-specific scores, but not the ALS-non-specific one (Table 2; Figure 2A–C). Indeed, patients harboring the GG genotype showed significantly lower scores compared to AA and AG carriers (ECAS total score median value 99 (35–123) in GG vs. 106 (31–129) in (AA + AG), *p* = 0.032; ECAS ALS-specific score median value 72 (22–91) in GG vs. 80 (21–97) in (AA + AG), *p* = 0.047) (Figure 2A,B). The difference in ECAS total and ALS-specific scores between the GG and (AA + AG) genotypes (i.e., under the recessive model) remained statistically significant after adjustment for age at onset, age at visit and survival [ECAS total score: F(1,191) = 6.068, *p* = 0.015; ECAS ALS-specific score: F(1,191) = 4.579, *p* = 0.034]. Conversely, no association with the ECAS total and ALS-specific scores was reported under the dominant and additive models (Table 2). Among the individual ECAS subdomains, however, a significant correlation was found only between the rs1990622 genotype and memory (i.e., one of the ALS-non-specific subdomains) under the recessive model (median value 14 (1–22) in GG vs. 16 (2–22) in (AA + AG), *p* = 0.050) (Table 2; Figure S2A–E). Like for the ECAS total and ALS-specific scores, the difference in the ECAS memory subdomain score between the GG and (AA + AG) genotypes was still significant after controlling for age at onset, age at visit, and survival [F(1,191) = 5.515, *p* = 0.020). No significant differences were detected by comparing cognitively impaired vs. unimpaired patients [i.e., (ALSci + ALScbi) vs. (ALScn + ALSbi)], as well as behaviorally impaired vs. unimpaired patients [i.e., (ALSbi + ALScbi) vs. (ALScn + ALSci)], according to the Strong criteria. Further, the distribution of ALS/FTD patients was similar across the different rs1990622 genotypes under all genetic models. Both the total score and the number of behavioral symptoms recorded at the ECAS carer interview, as well as the FBI total, FBI-A, and FBI-B scores, were not correlated with the rs1990622 genotype.

No significant correlation was found between the rs1990622 and age at onset, survival, and progression rate.

## 3. Discussion

In the last decade, the role of *TMEM106B* as a disease modifier of FTD has been widely investigated. An association between the rs1020004 genotype, which is in linkage disequilibrium with rs1990622, and disease duration was described by Van Deerlin and colleagues [16]. Specifically, shorter disease duration was found in patients homozygous for the major allele, which also increased disease susceptibility compared to the homozygotes for the minor one [16]. Neither the rs1020004 nor the rs1990622 genotypes affected age at onset in FTD patients [19,29], except for *GRN* mutation or *C9orf72* expansion carriers [25,29]. In this subset of patients, indeed, the risk allele of rs1990622 was linked to a reduced age at onset [25]. A recent GWAS performed on *GRN*-mutated FTD patients, however, failed to detect a significant association of the *TMEM106B* locus with age at onset [23]. Conversely, in FTD patients with *C9orf72* expansions, the risk allele was correlated with higher age at onset and age at death [29], although this association was not confirmed in the following studies [22,24]. The risk alleles of *TMEM106B* conferred an increased hazard of rapid cognitive decline in FTD patients, especially in the bvFTD subcategory [33]. 

In the general population, the risk allele of rs1990622 has been associated with reduced volume of brain regions involved in language functions (i.e., the gray matter of the left-sided superior temporal gyrus) and of the temporal lobe commissural tracts [34], whose atrophy belongs to the specific imaging pattern of FTD [35,36]. In line with these results, reduced brain connectivity within the left-sided frontoparietal (i.e., left precuneus) and the ventral salience (i.e., middle frontal gyrus) networks, whose involvement is typical of *GRN*-related FTD [37,38], was found at resting-state functional magnetic resonance imaging in non-demented *GRN* carriers [39].

The risk alleles of all the *TMEM106B* SNPs in linkage disequilibrium with rs1990622 have been associated with higher expression levels of the TMEM106B protein in the frontal cortex of FTD patients [16], albeit inconsistently [19]. This effect could be indirect and due to the presence of the coding SNP rs3173615 (p.T185S) within the aforementioned linkage disequilibrium block [26], which we also confirmed in our cohort. In previous studies, indeed, the presence of threonine at codon 185 (risk allele) rather than a serine (protective allele) was found to enhance TMEM106B protein levels independently from mRNA content because of a slower lysosomal degradation [26].

When starting from these data, the analysis of *TMEM106B*’s role as a disease modifier was rapidly extended to other neurodegenerative disorders [29]. The risk allele of rs1990622 predicted TPD-43 pathology both in AD patients and in elderly individuals without FTD [40,41]. Further, carriers of the minor rs1990622 allele were unlikely to develop hippocampal sclerosis [40,42,43], and this finding was subsequently confirmed also in Lewy Body dementia (LBD) patients [44]. When considering all AD patients independently from neuropathological alterations, the risk allele of rs1990622 conferred increased susceptibility to late-onset AD only in the apolipoprotein E (*APOE*) ε4 allele carriers [45], with no impact on cognitive deterioration progression [33]. Subsequently, however, a GWAS found an increased risk of AD associated with the major allele of rs1990622 only in females, independently of the *APOE* status [46]. The rs1990622 genotype did not influence the risk of Parkinson’s disease (PD) [30], multiple system atrophy (MSA) [30] and LBD [43], nor age at onset and decline of cognitive functions in PD/MSA [30]. In the following study, however, the rs1990622 risk allele was linked to a greater and faster cognitive deterioration in PD [33]. 

To date, few studies have focused on the impact of *TMEM106B* on disease susceptibility and genotype-phenotype correlation in ALS cohorts. The minor allele frequency detected in our cohort (0.40) is within the range of ALS populations in prior studies [22,31]. In ALS patients with and without *C9orf72* expansions, no association between the rs1990622 genotype and increased ALS susceptibility has been reported so far [22,29,31]. Age at death or onset apparently does not differ in ALS patients according to the rs1990622 genotype [22,29,30]. Independently of the *C9orf72* status, the disease duration and the burden of TDP-43 pathology are not influenced by *TMEM106B* SNP genotypes [31]. Our findings are in line with previous studies because we observed no significant differences in age at onset or survival amongst the different rs1990622 genotypes in our ALS cohort. 

In our work, we have shown that the major allele, traditionally considered the “risk” one, is associated with a higher frequency of bulbar site of onset. ALS with bulbar onset is usually characterized by reduced survival and worse quality of life [47,48,49]. Furthermore, to our knowledge, this is the first work that assesses the role of *TMEM106B* on both upper and lower motor neuron involvement in ALS. As regards the impact on upper motor neurons, we have shown that all patients harboring at least one major allele (AA + AG), and moreover, the homozygotes for the major allele (AA) have more upper motor neuron signs compared to the homozygotes for the minor allele (GG). As concerns lower motor neurons, instead, the presence of two major alleles seems to be protective against its deterioration, as AA carriers displayed significantly higher MRC total scores compared both to AG carriers and to all patients with at least one minor allele (AG + GG). Overall, the effects of the rs1990622 genotypes are opposite on upper and lower motor neuron involvement: whereas upper motor neuron signs predominate in those patients who carry at least one major risk allele (A), the lower motor neuron ones are higher in those who harbor at least one minor allele (G). Based on the ALSFRS-R scale, our findings indicate that the functional status of patients was more favorable in carriers of the GG genotype, who also showed the lowest burden of upper motor neuron signs according to the PUMNS, and worse in carriers of the AG genotype, who displayed a higher lower motor neuron involvement (i.e., lowest MRC total score). 

Results concerning the impact of *TMEM106B* on cognitive functions in ALS are debated in the literature. Although the minor allele of rs1990622 was associated with less cognitive impairment according to a phonemic verbal fluency test [31], the following study did not show significant differences in several cognitive domains’ performances (i.e., orientation/attention, memory, verbal fluency, language, and visuospatial ability) according to the rs1990622 genotypes [30]. Conversely, our work shows that the rs1990622 genotype has a strong impact on cognitive performances in ALS patients. Indeed, patients who are homozygous for the minor allele (GG genotype) showed lower ECAS total and ALS-specific scores (i.e., more severe cognitive impairment) compared to those who carry at least one major allele (both AA and AG genotypes). This difference remained significant even after controlling for factors that might have influenced the performance at neuropsychological tests, such as age at onset, age at visit, and survival, thus suggesting that *TMEM106B* has a strong impact on the cognitive profile of ALS patients. Compared to the previous study by Vass and colleagues, we have included a higher number of patients (195 vs. 61) who had been cognitively assessed, thus increasing the statistical power of our analysis [31]. Further, unlike previous studies, we have employed the ECAS, a neuropsychological multidomain assessment that has been specifically designed for ALS patients, whose Italian version proved to differentiate patients vs. controls [50]. In our cohort, the median ECAS total and ALS-specific scores of patients who were homozygous for the minor allele were abnormal (<105 and <77, respectively), whereas the sum of AA and AG carriers exceeded the cut-off scores on average. No significant differences were instead detected when testing the ALS-non-specific score. Intriguingly, however, the only ECAS subdomain which was significantly influenced by the rs1990622 genotype under the recessive model belonged to the ALS-non-specific ones, namely memory. Overall, our data suggest that the presence of two minor alleles of rs1990622 promotes a faster cognitive deterioration in ALS patients, especially concerning the combination of ALS-specific cognitive functions (i.e., language, verbal fluency, executive), but also memory. The presence of a higher burden of cognitive impairment among patients who are homozygous for the minor allele, traditionally considered “protective”, might be surprising. However, we should consider that the rs1990622 SNP does not represent a disease-causing variant per se but a common genetic modifier that regulates disease susceptibility and phenotypic heterogeneity by acting in concert with an unknown number of other genetic and environmental factors. In this scenario, at least two clues suggest that harboring two minor alleles of the rs1990622 SNP is a strong risk factor for faster cognitive deterioration associated with ALS, namely: (i) the consistent reduction of both ECAS total and ALS-specific scores in GG carriers; (ii) the exclusion of possible confounding factors, including age at visit (i.e., the higher rate of cognitive decline is not due to the presence of older subjects within the group of GG carriers), and site of onset. Indeed, previous investigations have identified a possible correlation between bulbar onset and ALS cognitive impairment [51,52], although inconsistently [53]. Here, however, we demonstrated that the proportion of patients with bulbar onset is significantly higher among carriers of the rs1990622 AA genotype, who did not show significantly worse performances at ECAS.

## 4. Materials and Methods

### 4.1. Participants and Clinical Assessment

A total of 865 patients who had been diagnosed with ALS and other motor neuron diseases (primary lateral sclerosis and progressive muscular atrophy) according to the El Escorial revised criteria [54] were recruited at IRCCS Istituto Auxologico Italiano between 2013 and 2021. The Ethics Committee of the Institute approved the study (18 May 2021). All participants gave written informed consent at the time of evaluation for using semi-anonymized clinical data for research purposes. The study was performed in accordance with the principles of the Declaration of Helsinki.

The following demographic and clinical variables were collected: sex, age at onset, age at diagnosis, site of onset, ALSFRS-R score at evaluation [55], and progression rate [calculated using the formula (48—ALSFRS-R score)/disease duration at evaluation expressed in months]. All patients were screened for mutations in the four main ALS-associated genes *C9orf72*, *SOD1*, *TARDBP*, and *FUS*. 

The PUMNS was used to explore the presence of upper motor neuron signs by evaluating both the bulbar segment (0–4 points) and the four limbs (0–7 points for each), with a total score ranging from 0 to 32 [56].

The burden of lower motor neuron involvement was explored by the MRC muscle scale. The MRC muscle scale attributes 0 to 5 points to three muscle groups for each limb (shoulder abductors, elbow flexors, wrist dorsiflexors, hip flexors, knee extensors, and ankle dorsiflexors), whose strength is compared to the maximum expected for that muscle (total score 0–60).

In a subset of 195 patients, we employed the ECAS—Italian version [50] to investigate cognitive functions. The ECAS total score is composed of an ALS-specific score (assessing the ALS-specific cognitive impairment, namely language, verbal fluency, and executive functions) and an ALS-non-specific score (assessing memory and visuospatial domains). Based on the performance at ECAS, patients were classified as ALScn, ALSbi, ALSci, or ALScbi according to the Strong revised criteria [57]. 

Further, in a subset of 155 patients, the presence of behavioral symptoms (disinhibition, apathy/inertia, loss of sympathy/empathy, perseverative/stereotyped/compulsive/ritualistic behaviors, and hyperorality/altered food preferences) was assessed by the ECAS carer interview and the FBI, which explores negative (FBI-A) and positive/disinhibited behaviors (FBI-B) [58]. 

Patients were classified as ALS/FTD when the diagnostic criteria for bvFTD [59] or for PPA [60] were met.

### 4.2. SNP Genotyping

Genomic DNA was extracted from peripheral blood using a commercial kit (Wizard Genomic DNA Purification Kit, Promega, Madison, WI, USA). SNP genotyping was performed using Human 660W-Quad BeadChips and Global Screening Arrays on HiScan platform (Illumina, San Diego, CA, USA) according to manufacturer’s instructions. Generated SNP data were then analyzed by GenomeStudio software (Illumina). Linkage disequilibrium between the rs1990622 and rs3173615 SNPs was assessed via the LDlinkR R package using GRCh37 as reference genome build.

### 4.3. Statistical Analysis

Statistical analyses were performed with the IBM Statistical Package for the Social Sciences (SPSS) version 26. Descriptive statistics were reported as medians and range for not normally distributed quantitative variables, or frequencies (%) for categorical variables. Comparison of the three rs1990622 genotypes was performed using three different genetic models: additive (AA vs. AG vs. GG), dominant [AA vs. (AG + GG)], and recessive [(AA + AG) vs. GG], where A and G are the major and minor alleles, respectively, and A represents the risk allele. Cross-tabulated frequencies and percentages were computed, and Chi-square or Fisher exact tests were performed to compare categorical variables, as appropriate. The non-parametric Kruskal–Wallis one-way analysis of variance (ANOVA) was employed to compare quantitative variables between two or more independent groups since the distribution of variables was not similar for all groups, as assessed by visual inspection of boxplots, and normality assumptions for parametric tests were not met according to the Kolmogorov–Smirnov normality test. When possible, post hoc analysis was performed to compare subgroups, including pairwise comparisons using Dunn’s test with a Bonferroni correction for multiple testing. Univariate Kaplan–Meier survival analysis with a log-rank comparison test was used to estimate the impact of the rs1990622 on survival. The non-parametric Quade’s rank analysis of covariance was run to determine the effect of the rs1990622 genotypes on ECAS scores after controlling for age at onset, age at visit (i.e., when ECAS was performed), and survival. Pairwise deletion was used to handle missing data.

## 5. Conclusions

Overall, our study demonstrates that *TMEM106B* is a strong disease modifier not only for FTD but also for ALS as well. The effects of the rs1990622, the top SNP associated with FTD in GWAS, encompass both cognitive and motor functions and, consequently, the functional status of ALS patients. Although we cannot explain how different *TMEM106B* genotypes result in such clinical heterogeneity in our ALS cohort, the characterization of disease subgroups with different phenotypic traits is a crucial requirement to develop targeted therapies and to guide patients’ enrollment in clinical trials. Indeed, previous studies have shown that the presence of cognitive and behavioral impairment is a known predictive factor of reduced survival in ALS [61,62] and that the genetic variability at disease susceptibility loci is a strong determinant of survival when administering novel treatments [63]. Future studies should therefore extend the analysis of *TMEM106B* to larger cohorts of ALS patients, especially if cognitively and behaviorally evaluated through specific neuropsychological scales and questionnaires, including the ECAS and the FBI. 

## Figures and Tables

**Table 1:** Demographic and clinical features, and genotype data of the ALS cohort.

Variable	N° Patients (Frequency)	Median (Range)
**Sex**	865	
**Male**	554 (64.0%)	
**Female**	311 (36.0%)	
**ALS family history**	865	
**familial**	20 (2.3%)	
**sporadic**	845 (97.7%)	
**ALS gene mutations**	865	
***C9orf72***	20 (2.4%)	
***SOD1***	0 (0.0%)	
***TARDBP***	4 (0.5%)	
***FUS***	2 (0.2%)	
**Age at onset (years)**	865	61.6 (13.6–90.6)
**Survival (months)**	865	27.5 (2.9–450.6)
**Site of onset**	815	
**Bulbar**	206 (25.3%)	
**Spinal**	609 (74.7%)	
**ALSFRS-R**	449	40 (9–48)
**PUMNS**	690	9 (0–29)
**MRC total score**	531	54 (6–60)
**ECAS total score**	195	105 (31–129)
**ECAS ALS-specific score**	195	78 (21–97)
**ECAS ALS-non-specific score**	195	27 (10–34)
**Cognitive phenotype (Strong revised criteria)**	195	
**ALScn**	87 (44.6%)	
**ALSbi**	35 (17.9%)	
**ALSci**	49 (25.1%)	
**ALScbi**	24 (12.3%)	
**N° of ALS/FTD patients**	25 (2.9%)	
**FBI total score**	155	2 (0–5)
**FBI A score**	155	1 (0–4)
**FBI B score**	155	0 (0–2)
**rs1990622 SNP genotype**	865	
**AA**	305 (35.3%)	
**AG**	421 (48.7%)	
**GG**	139 (16.1%)	

N°: number; ALS: amyotrophic lateral sclerosis; ALSFRS-R: ALS Functional Rating Scale Revised; PUMNS: Penn Upper Motor Neuron Score; MRC: Medical Research Council; ECAS: Edinburgh Cognitive and Behavioral ALS Screen; ALScn: cognitively normal ALS; ALSbi: behaviorally impaired ALS; ALSci: cognitively impaired ALS; ALScbi: cognitively and behaviorally impaired ALS; ALS/FTD: amyotrophic lateral sclerosis—frontotemporal dementia; FBI: Frontal Behavioral Inventory; SNP: single nucleotide polymorphism.

**Table 2:** Comparison of the site of onset, ALSFRS-R, PUMNS, MRC total score and ECAS scores amongst the *TMEM106B* rs1990622 genotypes under the genetic models employed.

	AA	AG	GG	(AG + GG)	(AA + AG)	Add. (AA vs. AG vs. GG)	Dom. [AA vs. (AG + GG)]	Rec. [(AA + AG) vs. GG]
	Median (Range)	Median (Range)	Median (Range)	Median (Range)	Median (Range)			
**Site of onset**	NA	NA	NA	NA	NA	0.074	**0.023**	0.340
**ALSFRS-R**	40 (9–48)	**39** **(16–48)**	**42** **(20–46)**	40 (16–48)	**39** **(9–48)**	**0.042**	0.471	**0.041**
**PUMNS**	**10** **(0–28)**	10 (0–29)	**7** **(0–29)**	9 (0–29)	**10** **(0–29)**	**0.025**	0.054	**0.015**
**MRC total score**	**55** **(14–60)**	**52** **(6–60)**	54 (12–60)	**53** **(6–60)**	54 (6–60)	**0.005**	**0.001**	0.424
**ECAS total score**	108 (31–129)	105 (39–128)	**99** **(35–123)**	104 (35–128)	**106** **(31–129)**	0.091	0.251	**0.032**
**ECAS ALS-specific score**	80 (21–97)	80 (24–95)	**72** **(22–91)**	77 (22–95)	**80** **(21–97)**	0.136	0.387	**0.047**
**ECAS ALS-non-specific score**	27 (10–34)	27 (13–34)	26 (11–34)	26 (11–34)	27 (10–34)	0.202	0.306	0.084
**ECAS memory score**	16 (2–22)	15 (2–22)	**14** **(1–22)**	15 (1–22)	**16** **(2–22)**	0.130	0.266	**0.050**

For each variable, the values which were statistically different are reported in bold (unadjusted *p* values). Add.: additive model; Dom.: dominant model; Rec.: recessive model; NA: not available; ALSFRS-R: ALS Functional Rating Scale Revised; PUMNS: Penn Upper Motor Neuron Score; MRC: Medical Research Council; ECAS: Edinburgh Cognitive and Behavioral ALS Screen; ALS: amyotrophic lateral sclerosis.

## Data Availability

The data presented in this study are archived on Zenodo (doi:10.5281/zenodo.6866601) and are available on request from the corresponding author.

## Supplementary Materials

The following supporting information can be downloaded at: https://www.mdpi.com/article/10.3390/ijms23169276/s1.
