# Supplementary material for: TMEM106B Acts as a Modifier of Cognitive and Motor Functions in Amyotrophic Lateral Sclerosis
Source: Int J Mol Sci. 2022 Aug 17;23(16):9276. doi: 10.3390/ijms23169276 (PMC9408885; doi:10.3390/ijms23169276)
Supplement: Supplementary file 1 [file ijms-23-09276-s001.zip › Figure S1.pdf]

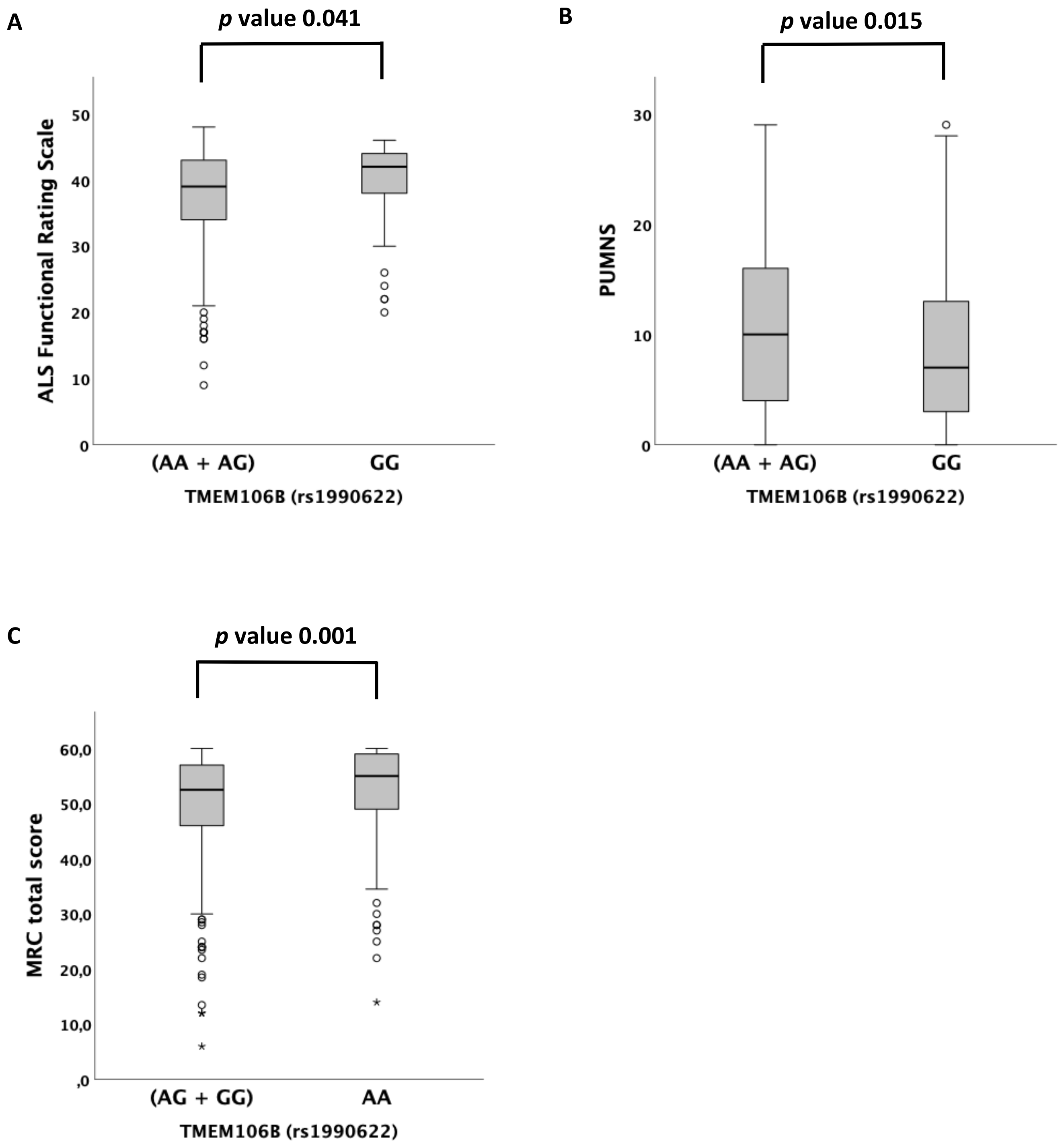

Figure S1. Distribution of variables amongst TMEM106B rs1990622 genotypes ac-cording to the Kruskal–Wallis one-way analysis of variance for independent samples un-der the dominant and/or recessive model. (**A**) ALSFRS-R (recessive). (**B**) PUMNS (recessive). (**C**) MRC total score (dominant). For each group, the bold line shows the median, the gray box represents the interquartile range (IQR) and whiskers show the 5° and 95° percentiles. Empty circles represent outliers.
