# Supplementary material for: TMEM106B Acts as a Modifier of Cognitive and Motor Functions in Amyotrophic Lateral Sclerosis
Source: Int J Mol Sci. 2022 Aug 17;23(16):9276. doi: 10.3390/ijms23169276 (PMC9408885; doi:10.3390/ijms23169276)
Supplement: Supplementary file 1 [file ijms-23-09276-s001.zip › Figure S2.pdf]

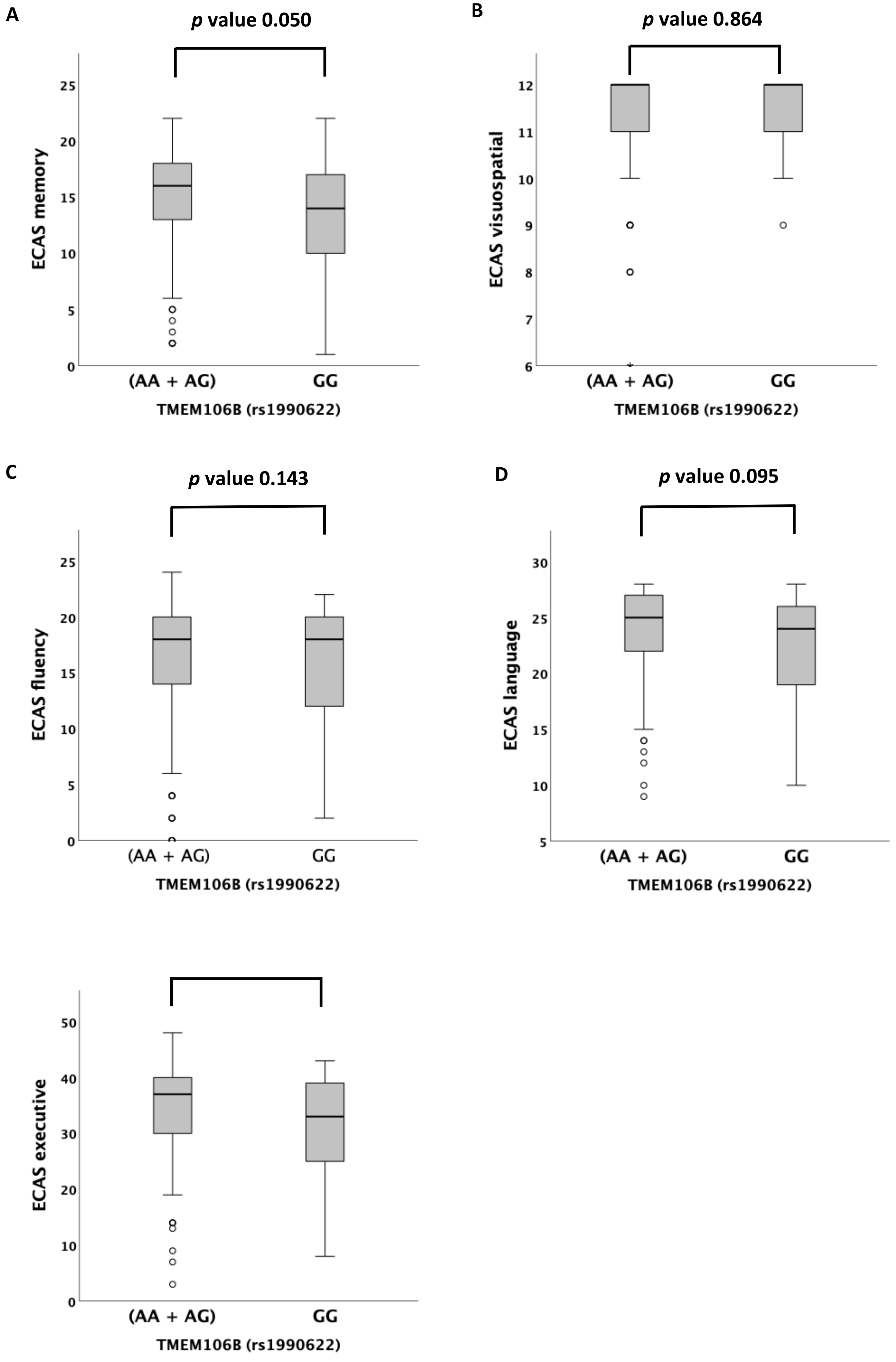

Figure S2. Distribution of ECAS subdomain scores amongst TMEM106B rs1990622 genotypes according to the Kruskal–Wallis one-way analysis of variance for independent samples under the recessive model. (**A**) Memory. (**B**) Visuospatial. (**C**) Fluency. (**D**) Language. (**E**) Executive. For each group, the bold line shows the median, the gray box represents the interquartile range (IQR) and whiskers show the 5° and 95° percentiles. Empty circles represent outliers.
