# Supplementary material for: TMEM106B Acts as a Modifier of Cognitive and Motor Functions in Amyotrophic Lateral Sclerosis
Source: Int J Mol Sci. 2022 Aug 17;23(16):9276. doi: 10.3390/ijms23169276 (PMC9408885; doi:10.3390/ijms23169276)
Supplement: Supplementary file 1 [file ijms-23-09276-s001.zip › Table S1.pdf]

Supplementary Table S1 – Comparison of the frequency of bulbar vs. spinal site of onset amongst the *TMEM106B* rs1990622 genotypes under a dominant model in the cohort of ALS patients.

		***TMEM106B* (rs1990622)**		**Total**
		**(AG + GG)**	**AA**	
**Site of onset**	**Bulbar**	120 (22.7%)	86 (30.0%)<br>(*p* value = 0.023)	206
	**Spinal**	408 (77.3%)<br>(*p* value = 0.023)	201 (70.0%)	609
**Total**		528	287	815

ALS: amyotrophic lateral sclerosis.
