# Supplementary material for: TMEM106B Acts as a Modifier of Cognitive and Motor Functions in Amyotrophic Lateral Sclerosis
Source: Int J Mol Sci. 2022 Aug 17;23(16):9276. doi: 10.3390/ijms23169276 (PMC9408885; doi:10.3390/ijms23169276)
Supplement: Supplementary file 1 [file ijms-23-09276-s001.zip › Table S2.pdf]

Table S2 – Comparison of the ALSFRS-R, PUMNS and MRC total score amongst the *TMEM106B* rs1990622 genotypes under the additive model before and after Bonferroni correction for multiple testing.

	**AA vs. AG**		**AA vs. GG**		**AG vs. GG**	
	**Unadjusted**	**Adjusted**	**Unadjusted**	**Adjusted**	**Unadjusted**	**Adjusted**
**ALSFRS-R**	0.139	0.418	0.237	0.712	**0.016**	**0.047**
**PUMNS**	0.230	0.690	**0.006**	**0.019**	0.055	0.164
**MRC total score**	**0.002**	**0.005**	**0.035**	0.106	**0.788**	**1.000**

For each variable, the values which were significantly different are reported in bold. Adjustment refers to the Bonferroni correction for multiple comparisons.
ALS: amyotrophic lateral sclerosis; ALSFRS-R: ALS Functional Rating Scale Revised; PUMNS: Penn Upper Motor Neuron Score; MRC: Medical Research Council; ECAS: Edinburgh Cognitive and Behavioral ALS Screen.
